# Myopia Control Efficacy of Grid Dimension Multiregion Spectacle Lenses: A One‐Year Randomized Double‐Masked Controlled Trial

**DOI:** 10.1155/joph/3220557

**Published:** 2026-02-24

**Authors:** Junfeng Wang, Weicong Lu, Ruru Chen

**Affiliations:** ^1^ National Clinical Research Center for Ocular Diseases, Eye Hospital, Wenzhou Medical University, Wenzhou, 325027, China

**Keywords:** axial length, myopia control, myopic defocus, spectacle lenses

## Abstract

**Purpose:**

To evaluate the effectiveness and safety of grid dimension multiregion (GDM) spectacle lenses in myopia control and compare the myopia control effects between GDM and highly aspherical lenslets (HAL) spectacle lenses.

**Methods:**

A total of 150 children aged 6–13 years were enrolled at the Hangzhou Campus of Eye Hospital of Wenzhou Medical University between December 2022 and July 2023 and randomly assigned to the GDM and HAL groups. Cycloplegic autorefraction (spherical equivalent refraction [SER]) and axial length (AL) were measured at baseline and 6‐month intervals until one year follow‐up.

**Results:**

131 participants completed the study and were included in the final analysis. At the six‐month follow‐up, the changes in SER and AL were 0.06 ± 0.37 D and 0.01 ± 0.11 mm in the GDM group and −0.06 ± 0.31 D and 0.04 ± 0.11 mm in the HAL group, respectively. The GDM group showed a statistically significant difference in SER changes compared to the HAL group (*p* = 0.042). No statistically significant difference in AL was observed between the groups (*p* = 0.266). At the one‐year follow‐up, the changes in SER were −0.17 ± 0.40 D in the GDM group and −0.19 ± 0.36 D in the HAL group. The changes in AL were 0.07 ± 0.17 mm in the GDM group and 0.11 ± 0.15 mm in the HAL group. No statistically significant differences in SER or AL were observed between the GDM and HAL groups (*p* = 0.980 and *p* = 0.131, respectively). After adjustment using generalized estimating equations, a significant difference in AL change was observed between the GDM and HAL groups (*p* = 0.048). In terms of subjective visual quality, there was no statistical difference in the total scores between the GDM and HAL groups (*p* = 0.436). No adverse events were reported in any group.

**Conclusions:**

Both GDM and HAL lenses effectively controlled myopia progression over 1 year, with GDM showing slightly superior efficacy in limiting axial elongation.

**Trial Registration**: Chinese Clinical Trial Registry: ChiCTR2200066890

## 1. Background

Myopia is the most prevalent refractive error, and its global prevalence has been steadily increasing in recent years [1, 2]. In China, the age of myopia onset has decreased significantly, and the proportion of adolescents with high myopia has also increased [3–5]. High myopia can lead to several complications, including posterior scleral staphyloma, choroidal neovascularization, retinal tears, and retinal detachment. These complications pose a serious threat to the visual health of patients with high myopia, often resulting in irreversible vision loss [6–8]. A previous study [9] found that a 1‐diopter increase in myopia is associated with a 67% increase in the prevalence of myopic maculopathy. Therefore, clinical interventions are essential to slow the progression of myopia.

Interventions including optical, pharmacological, and environmental approaches have been shown to effectively control the progression of myopia [10, 11]. Clinically, the primary optical methods for myopia control include orthokeratology, multifocal soft contact lenses, and specially designed spectacle lenses [12–14]. Numerous animal studies [15–17] found that myopic defocus inhibits the progression of myopia, whereas hyperopic peripheral defocus has the opposite effect. To reduce peripheral hyperopic defocus, these methods use various optical designs to induce myopic defocus in the peripheral retina. Previous studies [10–14] have demonstrated the effectiveness of these interventions in controlling myopia progression. Compared to the potential risks associated with contact lenses, spectacle lenses are more affordable and safer and are therefore often preferred [18].

Various designs of myopic defocus spectacle lenses have been developed, such as the defocus‐incorporated multiple segments (DIMS) spectacle lenses [14, 19], highly aspherical lenslets (HAL) spectacle lenses [20, 21], cylindrical annular refractive element (CARE) spectacle lenses [22], and lenslet‐array‐integrated (LARI) spectacle lenses [23]. Bao et al. [20] observed the myopia control effects of HAL lenses and found that, compared to single‐vision (SV) spectacle lenses, HAL lenses reduced myopia progression by 67% (0.53 *D*) and axial elongation by 64% (0.23 mm). These findings demonstrate the effectiveness of HAL lenses in myopia management. Liu et al. [22] developed a lens that incorporates high‐order aberrations while entirely excluding defocus components. Furthermore, research had shown that the new lens can slow myopia progression by 0.14 D and axial elongation by 0.09 mm, achieving effective myopia control. Both myopic defocus and high‐order aberrations have been shown to be effective in the prevention and control of myopia.

The grid dimension multiregion (GDM) spectacle lenses is a novel design consisting of a central circular optical zone for distance correction and 13 annular defocus zones. Each defocus zone contains microlenses that generate myopic defocus signals and microcylindrical lenses that produce high‐order aberrations. No studies have reported the effectiveness of the GDM design in myopia control.

Therefore, the primary aim of this study is to evaluate the effectiveness and safety of GDM lenses in controlling myopia. The secondary aim is to compare the myopia control efficacy of GDM and HAL lenses to determine whether any significant differences exist. These findings will provide valuable insights for informing clinical strategies in myopia management.

## 2. Methods

### 2.1. Study Design

This study was a prospective, randomized, double‐masked clinical trial. This study was scheduled to commence at the Hangzhou Campus of Eye Hospital of Wenzhou Medical University in December 2022. Follow‐up visits were scheduled every 6 months over a one‐year period. Adverse events (AEs) were assessed through clinical examinations at each follow‐up visit.

This study constitutes the third part of the larger research project, which was approved by the Ethics Committee of the Eye Hospital of Wenzhou Medical University (2022‐016‐K‐13‐02). Informed consent was obtained from the participants and their parents before enrollment, following a comprehensive explanation of the study’s potential risks. The trial protocol can be accessed in the Chinese Clinical Trial Registry. No protocol modifications were made throughout the trial.

No patients or public were involved in setting the research questions, defining outcome measures, or developing plans for recruitment, study design, or implementation. No patients were consulted regarding interpretation of results or manuscript preparation.

### 2.2. Eligibility Criteria

The inclusion criteria were as follows: age between 6 and 13 years; cycloplegic spherical equivalent refractive error between −0.75D and −5.00D; astigmatism ≤ 1.50D; anisometropia ≤ 1.50D; and best‐corrected visual acuity (BCVA) of 0.0 logMAR or better.

Exclusion criteria were as follows: history of ocular surgery; presence of strabismus, glaucoma, or other ocular diseases (excluding refractive errors); history of systemic diseases; current or prior use of myopia control interventions (low‐dose atropine eye drops, multifocal soft contact lenses, specially designed spectacle lenses, or orthokeratology lenses); and poor compliance or inadequate image quality.

### 2.3. Randomization and Masking

Computer‐generated randomization sequences were produced using Microsoft Excel 2019 (Microsoft Corp, Redmond, WA) by trained staff at Hangzhou Campus of the Eye Hospital of Wenzhou Medical University. A total of 150 participants were randomly assigned to the GDM group or the HAL group in a ratio of 1:1.

The interventions were prepared in sequentially numbered, opaque, and sealed packages. A single, unblinded research assistant generated the random sequence and assigned participants to groups. The research assistant was not involved in collecting or analyzing any data. All other investigators remained blinded throughout the trial. We prepared identical packages for both groups to maintain uniformity in appearance, color, and labels. Participants used opaque cases to store their spectacles during each follow‐up visit. The unblinded assistant was responsible for storing the spectacles and performing any adjustments and maintenance in a separate room. The blinded investigators were not permitted to handle or inspect the participants’ spectacles.

### 2.4. Intervention

Participants were assigned to wear GDM or HAL lenses. Participants were instructed to wear their assigned spectacle lenses daily for at least 12 h.

The HAL spectacle lenses feature a spherical front surface with 11 concentric rings composed of contiguous aspherical lenslets (each 1.1 mm in diameter). The nonlenslet area of the lens provides distance vision correction. The aspherical lenslet geometry is specifically designed to create a volume of myopic defocus anterior to the retina across all eccentricities, generating an optical signal for myopia control.

**FIGURE 1:**
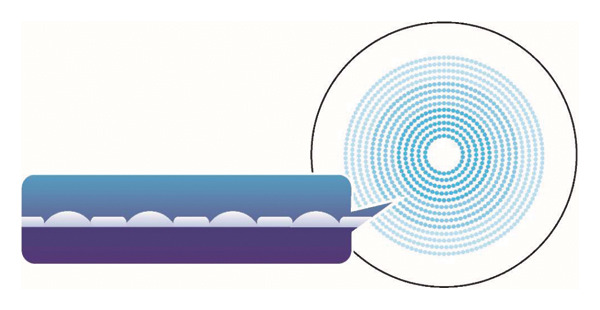
The design of the grid dimension multiregion (GDM) spectacle lens.

The GDM spectacle lenses (Figure 1) comprise a 9‐mm diameter circular central optical zone for clear distance vision correction and 13 surrounding annular defocus zones for myopia control. Within the defocus zones, a total of 1,040 microlenses and 1,040 microcylindrical lenses are embedded in a continuous, high‐density arrangement without intervening clear zones, guaranteeing a consistent defocus signal across the peripheral retina. This peripheral blur constitutes the intended optical mechanism for myopia control. The optical design parameters of the GDM lens are provided in Supporting Table S1.

### 2.5. Measurements

Cycloplegia was achieved using 3 drops of 1% cyclopentolate separated by 5 min. Measurements were performed 60 min after the third drop. Three measurements were performed using the Topcon KR‐800 autorefractor (Topcon Corporation, Tokyo, Japan). The mean value of the three measurements was recorded and used for analysis. Cycloplegia was performed at baseline and at the 6‐ and 12‐month follow‐up visits. Axial length (AL) was measured using the Tomey OA‐2000 optical biometer (Tomey Corporation, Nagoya, Japan), with 10 consecutive measurements taken. The device’s proprietary software automatically calculates the mean value after excluding the maximum and minimum readings from a series of repeated measurements to ensure reliability. All measurements were conducted by two experienced examiners.

#### 2.5.1. Subjective Visual Quality Assessment

Subjective vision quality was assessed using a structured questionnaire [24], which covered 10 common symptoms, with each symptom rated across three dimensions: frequency, severity, and bothersome. The questionnaire is included in the supporting information. A higher total score indicates worse subjective visual symptoms.

### 2.6. Sample Size

The sample size calculation was based on a superiority design. We chose an axial elongation difference of 0.045 mm between groups, which represents a 20% improvement over the HAL lens’s reported effect (0.23 mm). This difference was considered clinically meaningful by our investigators for evaluating a potential advancement in therapy. Based on the pilot study, the SD was 0.09 mm. A sample size of 57 participants per group provided 80% statistical power with a significance level (*α*) of 0.05. A minimum sample size of 144 individuals (72 per group) was needed, considering a 20% dropout rate.

### 2.7. Statistical Analysis

Only data from participants who completed all visits during the one‐year follow‐up were included in the analysis. The progression of myopia was evaluated through changes in spherical equivalent refraction (SER) and AL measurements. Data from baseline, six‐month, and one‐year follow‐up visits were analyzed. The mean values of ocular parameters measured in the right eye were used. Statistical analyses were performed using SPSS V26.0(IBM Corp, Armonk, NY). Independent sample *t*‐tests were used for normally distributed continuous variables, Mann–Whitney *U* tests for non‐normally distributed continuous variables, and chi‐squared tests for unordered categorical variables. Generalized estimating equations (GEEs) were conducted to assess the treatment effect, adjusting for baseline age, sex, baseline AL, or baseline SER. Pearson and Spearman correlation analyses were used to assess correlations between continuous variables. Multiple imputation was planned for the primary outcome measure in the case of missing data. A *p* value less than 0.05 was considered statistically significant.

### 2.8. AE Definitions and Reporting

An AE is any untoward medical occurrence, unintended disease or injury, or any unfavorable clinical sign (including an abnormal laboratory finding) in a subject, which may or may not be related to the investigational spectacle lenses. Any AE occurring during the study period, spontaneously reported by the subject or observed by others, will be recorded in the AE Form in the Case Report Form.

## 3. Results

### 3.1. Participants

**FIGURE 2:**
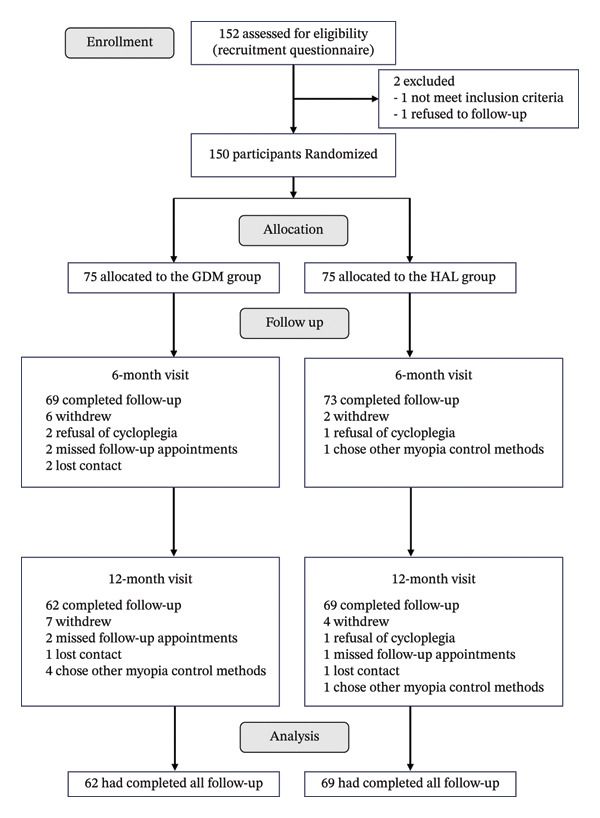
CONSORT diagram showing participant enrollment and retention. GDM = grid dimension multiregion spectacle lenses; HAL = highly aspherical lenslets spectacle lenses.

Eligible participants were recruited between December 2022 and July 2023. A total of 131 participants completed the one‐year follow‐up. Only data from the right eye were included in the analysis. Most participants received their assigned interventions according to the randomization protocol and demonstrated high adherence to the study regimen. Neither group received concomitant care during the trial period. Figure 2 shows the number of participants who were recruited and randomized, as well as the number who completed follow‐up visits. 19 participants withdrew from the study during the year (GDM group: *n* = 13; HAL group: *n* = 6). The reasons for withdrawal were as follows: refusal of cycloplegia (*n* = 4), missed follow‐up appointments (*n* = 5), lost contact (*n* = 4), and chose other myopia control methods (*n* = 6).

Table 1 presents the baseline demographic and clinical data. No statistically significant differences were observed among the groups at baseline. The GDM group consisted of 62 participants (27 males and 35 females), with a mean age of 10.13 ± 1.46 years. The HAL group comprised 69 participants (36 males and 33 females), with a mean age of 10.58 ± 1.48 years. At baseline, the SER and AL in the GDM group were −2.23 ± 0.89 D and 24.44 ± 0.70 mm, respectively. In the HAL group, the SER and AL were −2.24 ± 0.85 D and 24.56 ± 0.72 mm, respectively. No significant difference was found in baseline parameters. All enrolled participants met the inclusion criterion of a BCVA of 0.0 logMAR or better in each eye. This level of visual acuity was maintained at all scheduled follow‐up visits throughout the study period. All results are presented as the mean ± standard deviation unless otherwise specified.

**TABLE 1:** Baseline characteristics of participants who completed the 12 month visit.

Characteristics	GDM group (*n* = 62)	HAL group (*n* = 69)	*p* value
Age (years)	10.13 ± 1.46	10.58 ± 1.48	0.084^a^
Gender			0.324^b^
Male	27(43.5%)	36(52.2%)	
Female	35(56.5%)	33(47.8%)	
Cycloplegic autorefraction in SER (D)	−2.23 ± 0.89	−2.24 ± 0.85	0.766^c^
Axial length (mm)	24.44 ± 0.70	24.56 ± 0.72	0.344^a^

*Note:* Data are shown as mean ± SD in each group. GDM: grid dimension multiregion spectacle lenses. HAL: highly aspherical lenslets spectacle lenses. D = diopter.

Abbreviation: SER = spherical equivalent refraction.

^a^Independent samples *t*‐test.

^b^Chi‐squared test.

^c^Mann–Whitney *U* test.

### 3.2. Changes in SER

At the 6‐month follow‐up, the changes in SER were 0.06 ± 0.37 D for the GDM group and −0.06 ± 0.31 D for the HAL group. Statistically significant difference was observed between the GDM and HAL groups (*p* = 0.042). At the 12‐month follow‐up, the changes in SER for the GDM and HAL groups were −0.17 ± 0.40 *D* and −0.19 ± 0.36 *D*, respectively. No statistically significant difference was found between the GDM and HAL groups (*p* = 0.980) (Table 2 and Figure 3(a)).

**TABLE 2:** Unadjusted mean ± standard deviation changes in SER and AL in each group.

Variable	GDM group (*n* = 62)	HAL group (*n* = 69)	*p* value	Difference (95% CI)
Change in SER (D)				
6 months	0.06 ± 0.37	−0.06 ± 0.31	0.042^a^	0.12 (0.004 to 0.244)
12 months	−0.17 ± 0.40	−0.19 ± 0.36	0.98^b^	0.02 (−0.125 to 0.125)
Change in AL (mm)				
6 months	0.01 ± 0.11	0.04 ± 0.11	0.266^b^	−0.03 (−0.060 to 0.020)
12 months	0.07 ± 0.17	0.11 ± 0.15	0.131^a^	−0.04 (−0.097 to 0.013)

*Note:* Data are shown as mean ± SD in each group. D = diopter; GDM: grid dimension multiregion spectacle lenses. HAL: highly aspherical lenslets spectacle lenses.

Abbreviations: AL = axial length, SER = spherical equivalent refraction.

^a^Independent sample *t*‐test.

^b^Mann‐Whitney *U* test.

FIGURE 3Unadjusted mean change in SER (a) and AL (b) over 12 months. GDM = grid dimension multiregion spectacle lenses; HAL = highly aspherical lenslets spectacle lenses; SER = spherical equivalent refraction; D = diopters; AL = axial length. Error bars represent the standard error.

**Figure:**
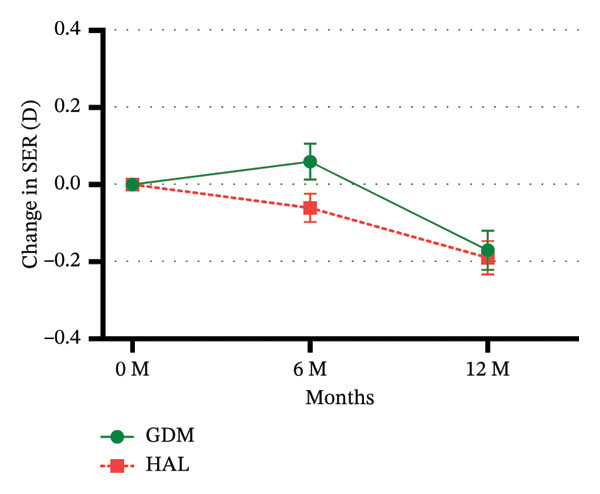
(a)

**Figure:**
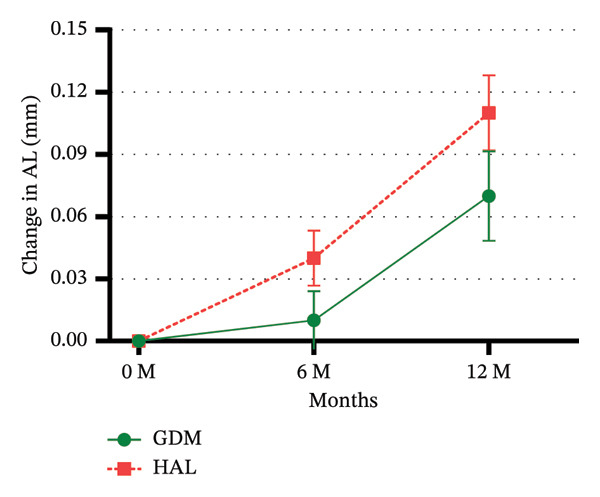
(b)

In the GEE analysis, no covariates were significantly associated with changes in SER. After adjustment, the mean 6 month SER changes in the GDM and HAL groups were 0.06 ± 0.05 D and −0.06 ± 0.04 *D*, respectively. The mean 12 month SER changes in the GDM and HAL groups were −0.17 ± 0.05 D and −0.19 ± 0.04 *D*, respectively. There was still no statistically significant difference in SER changes between the GDM and HAL groups (*p* = 0.226). A statistically significant interaction effect between lens type and time was observed (*p* = 0.040) (Table 3). Following stratified analysis, the outcomes were consistent with unadjusted results. Pearson’s correlation analysis showed that age was not significantly correlated with changes in SER in either the HAL (*ρ* = 0.047; *p* = 0.699) or GDM groups (*r* = 0.025; *p* = 0.849) (Table 4 and Figure 4(a)).

**TABLE 3:** Results of the generalized estimating equations.

Factors	SER change			AL change		
	β	*p* value^a^	95% CI	β	*p* value^a^	95% CI
Group (GDM, HAL)	0.071	0.226	−0.044 to 0.186	0.044	0.048	0.000 to 0.087

*Covariates*						
Gender	−0.026	0.653	−0.137 to 0.086	0.006	0.778	−0.037 to 0.049
Baseline age (years)	0.004	0.855	−0.035 to 0.043	−0.024	< 0.001	−0.037 to −0.011
Baseline SER (D)	−0.040	0.208	−0.103 to 0.022	—	—	—
Baseline AL (mm)	—	—	—	0.017	0.205	−0.010 to 0.044
Time	0.178	< 0.001	0.127 to 0.230	0.064	< 0.001	0.049–0.079
Time∗group	0.105	0.040	0.005 to 0.206	0.014	0.343	−0.015 to 0.044
Group∗baseline age	−0.028	0.440	−0.099 to 0.043	0.013	0.307	−0.012 to 0.037

*Note:* D = diopter; GDM: grid dimension multiregion spectacle lenses. HAL: highly aspherical lenslets spectacle lenses.

Abbreviations: AL = axial length, SER = spherical equivalent refraction.

^a^Generalized estimating equations.

**TABLE 4:** Correlation between baseline age and myopia progression in each group.

**Correlation coefficient (age—myopia progression)**						
	**12 months SER change**			**12 months AL change**		
	***r* or ρ**	*p* **value**	**95% CI**	***r* or ρ**	*p* **value**	**95% CI**

GDM	0.025	0.849^a^	−0.226 to 0.273	−0.189	0.142^a^	−0.419 to 0.064
HAL	0.047	0.699^b^	−0.198 to 0.287	−0.343	0.004^a^	−0.536 to −0.115

*Note:* GDM: grid dimension multiregion spectacle lenses. HAL: highly aspherical lenslets spectacle lenses.

Abbreviations: AL = axial length, SER = spherical equivalent refraction.

^a^Pearson correlation test.

^b^Spearman correlation test.

FIGURE 4Correlation between baseline age and change in SER (a) and AL (b) in each group. GDM = grid dimension multiregion spectacle lenses; HAL = highly aspherical lenslets spectacle lenses; SER = spherical equivalent refraction; D = diopters; AL = axial length.

**Figure:**
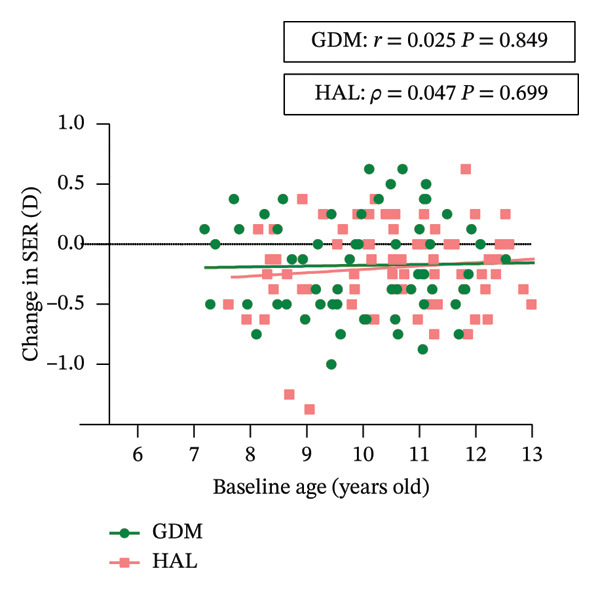
(a)

**Figure:**
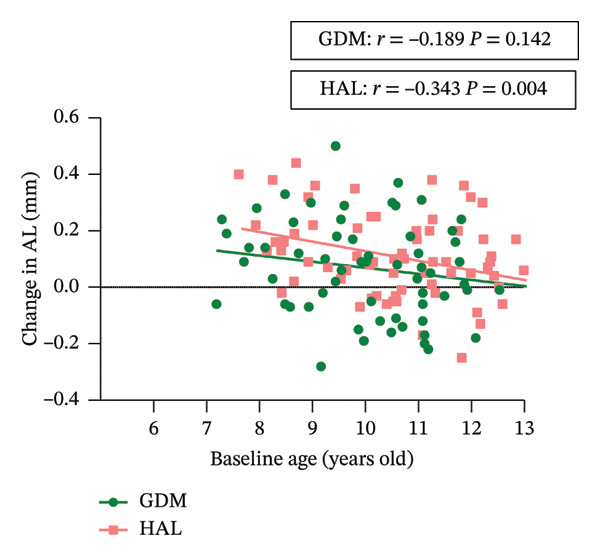
(b)

### 3.3. Changes in AL

The 6‐month follow‐up showed that changes in AL were 0.01 ± 0.11 mm for the GDM group and 0.04 ± 0.11 mm for the HAL group. No statistically significant difference was observed between the GDM and HAL groups (*p* = 0.266). After 12‐month of follow‐up, the changes in AL for the GDM and HAL groups were 0.07 ± 0.17 mm and 0.11 ± 0.15 mm, respectively. There was no statistically significant difference between the GDM and HAL groups (*p* = 0.131) (Table 2 and Figure 3(b)).

In the GEE analysis, baseline age (*p* = 0.001) was significantly associated with AL progression. After adjustment, the mean 6‐month AL changes in the GDM and HAL groups were 0.01 ± 0.01 mm and 0.04 ± 0.01 mm, respectively. The mean 12‐month AL changes in the GDM and HAL groups were 0.06 ± 0.02 mm and 0.11 ± 0.02 mm, respectively. A significant difference in the AL change was observed between the GDM and HAL groups at the one‐year follow‐up (*p* = 0.048). The interaction between group and baseline age was tested in the GEE model and was not statistically significant (*p* = 0.307) (Table 3). Pearson’s correlation analysis showed that age was significantly correlated with changes in AL in the HAL group (*r* = −0.343; *p* = 0.004) but not in the GDM group (*r* = −0.189; *p* = 0.142) (Table 4 and Figure 4(b)).

### 3.4. Distribution of Participants With Myopia Progression

Approximately 41.9% of participants in the GDM group and 37.7% in the HAL group exhibited no SER increase (≤ 0 D change from baseline) during the 12‐month study. 37.1% and 24.6% of participants in the GDM and HAL groups showed no AL elongation (≤ 0 mm change from baseline) during the study period. These results are presented in Figure 5.

FIGURE 5The distribution of myopia progression based on the 12‐month change in SER (a) and AL (b). GDM = grid dimension multiregion spectacle lenses; HAL = highly aspherical lenslets spectacle lenses; SER = spherical equivalent refraction; D = diopters; AL = axial length.

**Figure:**
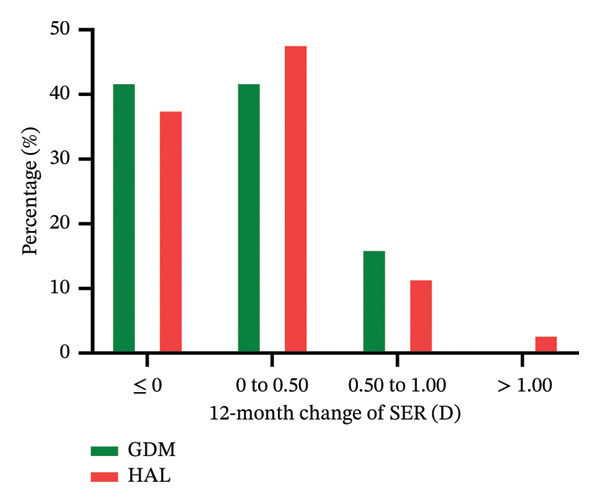
(a)

**Figure:**
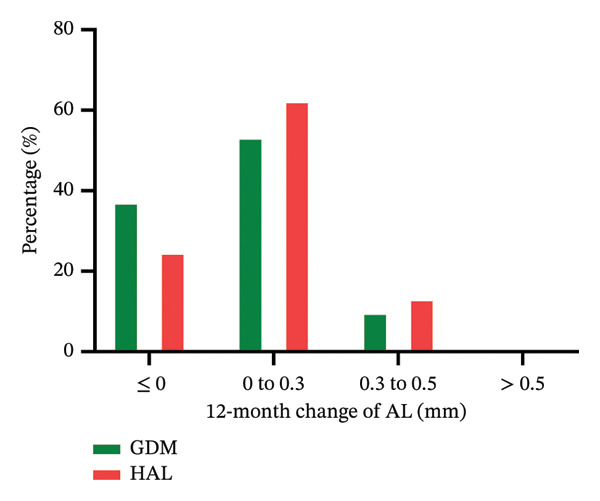
(b)

### 3.5. Subjective Visual Quality

No significant difference was found in the total score between the GDM and HAL groups (*p* = 0.436). The median score was 2 (IQR: 0–5) for GDM and 0 (IQR: 0–4) for HAL. The distribution of total scores across intervals is presented in Table 5. The majority of participants in both groups reported minimal symptoms (scores of 0 or 1–5).

**TABLE 5:** Subjective visual quality questionnaire scores in each group.

**Core statistical results**	***Z* value**	**p**value**^a^**	**GDM group median (IQR)**	**HAL group median (IQR)**
**Total Score**	**−0.778**	**0.436**	**2 (0–5)**	**0 (0–4)**

**Score distribution [*n* (%)]**			**GDM group (*n* = 62)**	**HAL group (*n* = 69)**

Total score				
0 points			29 (46.8)	36 (52.2)
1–5 points			18 (29.0)	18 (26.1)
6–10 points			11 (17.7)	12 (17.4)
> 10 points			4 (6.5)	3 (4.3)

*Note:* GDM: grid dimension multiregion spectacle lenses. HAL: highly aspherical lenslets spectacle lenses.

^a^Mann–Whitney *U* test.

### 3.6. AEs

No AEs were reported in either group during the one‐year follow‐up period.

## 4. Discussion

In recent years, the annual incidence of myopia has increased, characterized by earlier onset, rapid progression, and a higher proportion of high myopia, particularly during the growth and development stages of children. With growing clinical demand for myopia prevention and control, a wide variety of myopia defocus spectacle lens designs have emerged, making evidence‐based guidance on lens selection increasingly critical.

Based on the established efficacy of myopia control lenses and for ethical considerations, we decided not to include the SV control group. The treatment effects of the GDM and HAL lenses were evaluated independently. Our observed one‐year changes with HAL lenses (−0.19 *D*, 0.11 mm) were consistent with the Bao et al. trial (−0.27 *D*, 0.13 mm) [20]. In our cohort, GDM lens wear resulted in mean progressions of −0.17 D (SER) and 0.07 mm (AL) over one year.

GDM lenses showed greater efficacy in SER control at the six‐month follow‐up. However, no statistically significant difference was found between GDM and HAL lenses after one year. The findings show that GDM lenses control SER better in the early stages. However, their effectiveness decreases over time. This matches observations from prior research on long‐term optical interventions [25–30]. The trend is consistent with the neurophysiological concept of blur adaptation [31]. A study indicated that the efficacy of two types of myopia control spectacle lenses decreased from approximately 60%–80% initially to 35%–55% over a two‐year period [27]. The gradual neural adaptation to the constant peripheral defocus signal serves as a compensatory mechanism, diminishing the intervention’s effect. The adaptive response appears strong enough to counteract the beneficial trend of age‐related myopia slowing. Consequently, despite this natural slowing, we did not observe the anticipated stabilization or improvement in efficacy over time. As a result, the observed outcome is a reduction in control efficacy noted in our study and others. Thus, comprehending and addressing blur adaptation could be crucial for enhancing myopia control. Future research needs to overcome the visual system’s strong adaptive capacity. Securing long‐term efficacy will require a dual strategy: enhancing the strength of static stimuli while also innovating with dynamic and unpredictable optical strategies.

In terms of AL control, the analysis suggested a potential advantage of GDM lenses over HAL lenses in controlling AL, though this difference was of borderline statistical significance (*p* = 0.048). While animal models and some clinical studies suggest a dose‐dependent relationship between defocus strength and efficacy [32–35], our results indicate that this relationship may be nonlinear in humans over one year. Optically, the GDM lens operates within a higher range of myopic defocus (5.0–6.0 *D*), compared to the HAL lens (3.5–5.0 *D*). A previous study [22] has demonstrated the effectiveness of lenses utilizing microcylindrical lenses in myopia control. The optical principle of this design is that the microcylindrical lenses generate complex wavefront perturbations that are interpreted as extreme high‐order aberrations, which contribute to the retinal blur signal. The GDM lens incorporates similar, but more densely packed, microcylindrical lenses (with a power of + 8.00 to + 9.00 *D*) designed to induce analogous higher‐order aberrations, in addition to the continuous myopic defocus from its annular microlenses. We hypothesize that this combined optical strategy may contribute to the preliminary findings of a slightly greater efficacy in AL control compared to the HAL lens, as suggested by the observed trend (*p* = 0.048). However, the considerable optical enhancement resulted in only a marginal clinical benefit. This suggests the possibility of diminishing returns, where incremental gains in efficacy decrease after a certain stimulus threshold. The HAL may already be approaching this threshold. This overall mechanism requires validation through future studies specifically designed to measure and quantify the optical property and relative contribution of these different visual signal mechanisms.

When compared with results from other published trials, the absolute changes observed with GDM lenses in our cohort appear numerically favorable relative to those of other designs, such as PLARI (−0.30 *D*, 0.19 mm) [23] and CARE (−0.56 *D*, 0.26 mm) [22], and were similar to those reported for DIMS lenses (−0.17 *D*, 0.11 mm) [14]. However, these numerical comparisons must be interpreted with caution due to important differences in study cohorts and settings. For instance, the geographic and environmental context of our study (Hangzhou, Zhejiang) differs from that of the cited trials conducted in Hong Kong (DIMS) and Wenzhou, Zhejiang (PLARI, CARE), which may influence myopia progression rates. Our enrolled age range (6–13 years) was comparable to that of other studies (PLARI, DIMS, CARE). However, variations in study methods and patient baselines hinder any valid direct comparison. Consequently, comparing our research with the existing literature merely provides context.

The study revealed a significant association between AL change in the HAL group and age, while no such association was identified in the GDM group. Nonetheless, the formal test for interaction between the treatment group and age did not yield statistically significant results. Consequently, our research lacks adequate evidence to suggest an optimal age threshold for the selection between GDM and HAL lenses. The within‐group correlations observed should be regarded as exploratory and necessitate further validation in larger studies. A significant observation was the dissociation between the correlation of age with AL change and SER change within the HAL group, despite a general correlation between the two measures. This discrepancy may be attributed to physiological differences. AL is an objective biometric indication of structural change. As a net refractive result, SER combines AL and anterior segment optical power. It is well known that the crystalline lens undergoes compensatory changes to reduce the refractive impact of axial elongation [36]. Therefore, the significant correlation with AL change points to an age‐related effect on structural progression. But as there is no similar correlation with SER change, this structural effect does not dependably lead to the refractive outcome. Thus, AL is a more accurate indicator for evaluating the effectiveness of myopia control.

Our findings contribute to a body of literature showing an inconsistent relationship between age and myopia control effectiveness. Lam et al. found a significant correlation between age and SER within the DIMS group [14]. Jiang et al. identified significant correlations between changes in both SER and AL in the PLARI group and age [23]. Conversely, Bao et al. found no correlation between changes in SER or AL and age within the HAL group [20]. Considering the similar optical design of the HAL lenses used, this indicates that other methodological or population factors could be accountable, such as choice of comparator groups, participant age range, regional environmental risks, and sample size.

In terms of subjective visual quality, this study found no statistical difference in the total scores between the GDM and HAL groups. Both groups had low median scores. The impact on visual quality was minor. Most children exhibited good tolerance to both lenses.

There are some limitations with this study. The assessment of visual quality was based on subjective reports and lacked objective evaluation, including tests of contrast sensitivity and modulation transfer function. Adding these assessments would have allowed for a more robust evaluation of performance. Our discussion regarding the role of higher‐order aberrations in the GDM lenses mechanism is based on optical inference from the lens microstructure and cited literature, rather than on direct wavefront measurements of the lenses themselves. Future studies incorporating quantitative optical analysis, such as wavefront sensing, would be valuable to directly characterize and compare the HOA profiles of different myopia control lenses designs. The influence of daily wear time, near work, and outdoor activities on myopia progression was not quantified in this study, and the use of questionnaires may introduce recall bias. Future studies would benefit from supplementing questionnaires with objective data collected by wearable devices. The study did not include an SV control group; we cannot determine the absolute treatment effect of the GDM and HAL lenses. Future studies incorporating an SV control group are needed to further evaluate their myopia control efficacy.

## 5. Conclusion

In summary, this study demonstrates that both GDM and HAL spectacle lenses provided effective myopia control over a one‐year follow‐up period, with no serious AEs reported. However, further long‐term follow‐up studies are needed to evaluate the sustained efficacy of these lenses in myopia control.

**Nomenclature**
GDM: Grid Dimension Multiregion
HAL: Highly aspherical lenslets
SER: spherical equivalent refraction
AL: axial length
DIMS: defocus‐incorporated multiple segments
CARE: cylindrical annular refractive element
LARI: lenslet‐array‐Integrated
SV: single‐vision
GEE: Generalized Estimating Equations
BCVA: best‐corrected visual acuity

## Author Contributions

Junfeng Wang and Ruru Chen conceived and designed the study; Junfeng Wang executed the experimental protocols and drafted the manuscript; statistical analyses were performed by Junfeng Wang and Weicong Lu; Junfeng Wang and Ruru Chen collected clinical data and conducted follow‐up assessments.

## Funding

This study was supported by program‐specific funding from the Medical Research Excellence Program of the Eye Hospital of Wenzhou Medical University (7‐10‐2023‐%s‐0003).

## Disclosure

The funders had no role in study design, data collection and analysis, decision to publish, or preparation of the manuscript. All authors read and approved the final manuscript.

## Ethics Statement

The study received ethics approval from the Ethics Committee of the Eye Hospital of Wenzhou Medical University, adhering to the Declaration of Helsinki guidelines. Written informed consent was secured from all participants and their legal guardians, including authorization for anonymous data publication.

## Consent

This study was conducted and reported in accordance with the Consolidated Standards of Reporting Trials (CONSORT) 2025 guidelines.

## Conflicts of Interest

The authors declare no conflicts of interest.

## Supporting Information

Additional supporting information can be found online in the Supporting Information section.

## Supporting information


**Supporting Information 1** Supporting Table S1. Detailed optical design parameters of the GDM spectacle lenses evaluated in this study.

**Supporting Information 2** Supplementary Questionnaire (The Quality of Vision (QoV) Questionnaire). The standardized instrument used in this study to assess subjective visual quality across 10 symptoms, rated by frequency, severity, and bothersome.

## Data Availability

The datasets used and analyzed during the current study are available from the corresponding author on reasonable request.
