# Supplementary material for: Myopia Control Efficacy of Grid Dimension Multiregion Spectacle Lenses: A One‐Year Randomized Double‐Masked Controlled Trial
Source: J Ophthalmol. 2026 Feb 24;2026:3220557. doi: 10.1155/joph/3220557 (PMC12930210; doi:10.1155/joph/3220557)
Supplement: Supplementary file 1 — Supporting Information 1 Supporting Table S1. Detailed optical design parameters of the GDM spectacle lenses evaluated in this study. [file JOPH-2026-3220557-s001.docx]

Table S1. Details of the GDM spectacle lenses

| Design | Details |
| --- | --- |
| The diameter of the central optical zone | 9mm |
| The innermost circle center distance | 10 ± 0.05 mm |
| the outermost circle center distance | 52 ± 0.1 mm |
| the center distance between adjacent micro-lenses in the same circle | 1.22 ± 0.05 mm |
| the center distance between lenses in adjacent micro-lenses | 1.75 ± 0.05 mm |
| the line spacing between adjacent micro-cylindrical lenses | 0.2 mm |

GDM: Grid Dimension multi-region spectacle lenses.
