# Supplementary material for: Myopia Control Efficacy of Grid Dimension Multiregion Spectacle Lenses: A One‐Year Randomized Double‐Masked Controlled Trial
Source: J Ophthalmol. 2026 Feb 24;2026:3220557. doi: 10.1155/joph/3220557 (PMC12930210; doi:10.1155/joph/3220557)

**The Quality of Vision (QoV) Questionnaire**

**Frequency Never (0) Occasionally (1) Quite often (2) Very often (3)**

**Severity Not at all (0) Mild (1) Moderate (2) Severe (3)**

**Bothersome Not at all (0) A little (1) Quite (2) Very (3)**

**1.** **How often do you experience glare?**

**How severe is the glare?**

**How bothersome is the glare?**

**2. How often do you experience haloes?**

**How severe are the haloes?**

**How bothersome are the haloes?**

**3. How often do you experience starbursts?**

**How severe are the starbursts?**

**How bothersome are the starbursts?**

**4. How often do you experience hazy vision?**

**How severe is the hazy vision?**

**How bothersome is the hazy vision?**

**5. How often do you experience blurred vision?**

**How severe is the blurred vision?**

**How bothersome is the blurred vision?**

**6. How often do you experience distortion?**

**How severe is the distortion?**

**How bothersome is the distortion?**

**7. How often do you experience double or multiple images?**

**How severe are the double or multiple images?**

**How bothersome are the double or multiple images?**

**8. How often do you experience a fluctuation in your vision?**

**How severe is the fluctuation in your vision?**

**How bothersome is the fluctuation in your vision?**

**9. How often do you experience focusing difficulties?**

**How severe are the focusing difficulties?**

**How bothersome are the focusing difficulties?**

**10. How often do you experience difficulty judging distance or depth perception?**

**How severe is the difficulty judging distance or depth perception?**

**How bothersome is the difficulty judging distance or depth perception?**


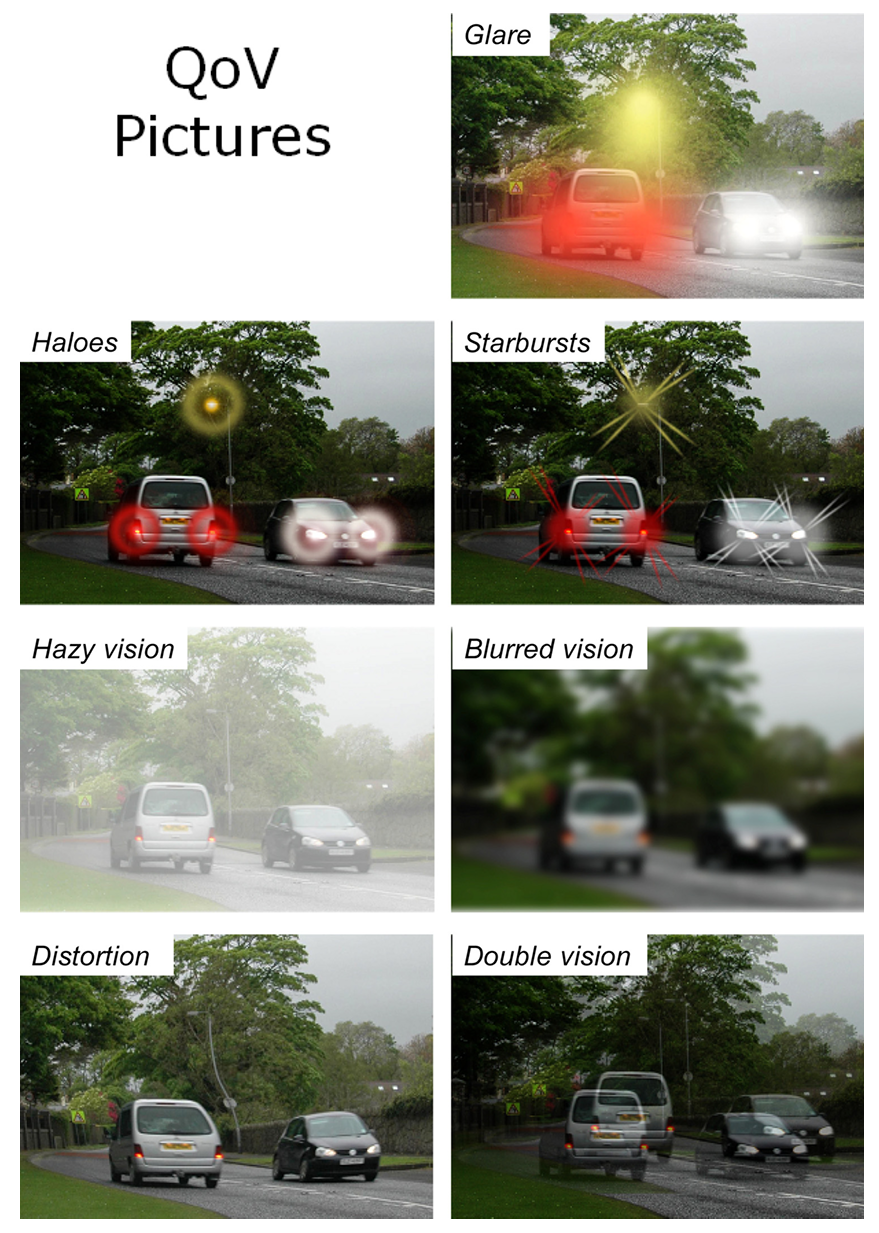

Supplement: Supplementary file 2 — Supporting Information 2 Supplementary Questionnaire (The Quality of Vision (QoV) Questionnaire). The standardized instrument used in this study to assess subjective visual quality across 10 symptoms, rated by frequency, severity, and bothersome. [file JOPH-2026-3220557-s002.docx]
